# From unicuspid to quadricuspid: the impact of aortic valve morphology on 3D hemodynamics

**DOI:** 10.1186/1532-429X-15-S1-O79

**Published:** 2013-01-30

**Authors:** Pegah Entezari, Susanne Schnell, Riti J Mahadevia, Daniel Rinewalt, Amir H Davarpanah, SC Malaisrie, Patrick McCarthy, Jeremy Collins, James Carr, Michael Markl, Alex J Barker

**Affiliations:** 1 Radiology, Northwestern University, Chicago, IL, USA; 2 Northwestern University, Chicago, IL, USA

## Background

The purpose of this study was to assess the impact of aortic valve morphology on aortic 3D blood flow dynamics and wall shear stress (WSS) in normal and a wide range of congenitally altered aortic valves ranging from unicuspid to quadricuspid morphology.

## Methods

In-vivo aortic 3D hemodynamics were evaluated by MRI in 17 patients with unicuspid (n=3), bicuspid (n=9, 3 true bicuspid, 3 right-left (RL) coronary leaflet fusion, and 3 right-non (RN) coronary leaflet fusion), trileaflet (n=3), and quadricuspid aortic valves (n=2). Valve morphology and dynamics were assessed using 2D CINE MRI. Aortic blood flow was measured using ECG and respiration synchronized 4D flow MRI with full volumetric coverage of the aorta. Data analysis included co-registered visualization of aortic valve morphology with systolic 3D blood flow and grading of valve-specific aortic out-flow patterns. The influence of valve geometry on aortic hemodynamics was quantified by calculation of valve flow angle and segmental distribution of ascending aorta WSS.

## Results

All congenitally altered valves showed marked flow derangement, elevated velocity jets along the aortic wall and distinct flow impingement locations. While all RL-BAV valves were associated with flow jets directed towards the right anterior aortic wall, RN-fusion and unicuspid valves showed jet patterns towards the right-posterior wall. Quantitative analysis revealed higher flow angles for unicuspid, true BAV, RN-BAV, RL-BAV, and quadricuspid valves (θ= 47±10, 28±2, 29±18, 18±12, 15±2) compared to controls (θ=10±6). In addition, WSS eccentricity was increased and variable between valve groups ( 0.25 N/m^2^, 0.23 N/m^2^, 0.29 N/m^2^, 0.44 N/m^2^, and 0.36 N/m^2^ for unicuspid, true BAV, RN-BAV, RL-BAV, and quadricuspid) compared to controls (0.07 N/m^2^) indicating differences in regional wall stress despite similar average WSS (0.5±0.1 N/m2, 0.5±0.1 N/m^2^, 0.6±0.3 N/m^2^, 0.5±0.1 N/m^2^, and 0.5±0.1 N/m^2^ in unicuspid, true BAV, RN-BAV, RL-BAV, and quadricuspid valves) compared to controls (0.4±0.2 N/m^2^). The qualitative assessment of jet flow impingement region corresponded to the quantitative location of regionally elevated peak WSS.

## Conclusions

Changes in aortic hemodynamics are closely linked with differences in congenitally altered valve morphology. Future studies in larger cohorts and longitudinal follow-up are warranted to evaluate the implications for the development and progression of valve morphology specific aortic disease.

## Funding

Grant support by NIH R01HL115828, NUCATS Dixon Award.

**Table 1:** Demographics and flow quantification

	Unicuspid	Bicuspid			Trileaflet	Quadricuspid
		
		True	Right-none	Right-left		
n (female)	3(1)	3(2)	3(2)	3(0)	3(1)	2(1)

Age	43.1±4.0	39.1±2.9	42.2±10.8	51.9±9.1	38.7±5.5	40.6±0.5

AAo Diameter (cm)	3.9±0.6	3.4±0.1	3.4±0.7	3.9±0.5	2.4±0.5	3.5±1.1

Flow Angle(°)	47±10	28±2	29±18	18±12	10±6	15±2

Flow Imp. (A,R,P,L)	RP,P,P	RA,RP,-	RP,P,P	A,RA,RA	-,-,-	RA,-

Mean WSS (N/m^2^)	0.5±0.1	0.5±0.1	0.6±0.3	0.5±0.1	0.4±0.2	0.5±0.1

Peak WSS (N/m^2^)	0.6±0.2	0.6±0.3	0.7±0.5	0.7±0.2	0.5±0.1	0.7±0.2

Peak WSS location	P	RP	P	RA	RA	LA

WSS Ecc. (N/m^2^)	0.25	0.23	0.29	0.44	0.07	0.36

R: Right, L: Left, A: Anterior, P: Posterior

**Figure 1:**
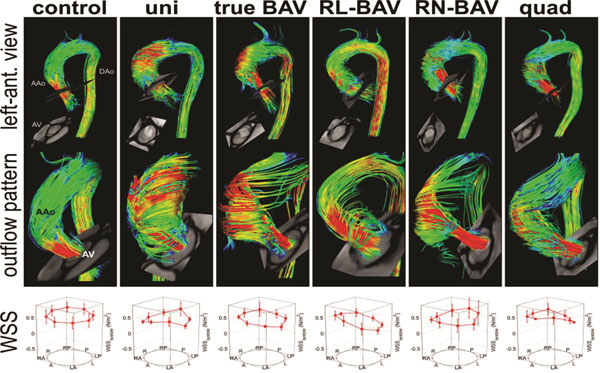
Relationships between valve morphology, 3D flow patterns in ascending aorta distal to the aortic valve (AV) and segmental systolic WSS distribution. The segmental WSS distribution represents the average over all subjects for each type of leaflet morphology. Note the different systolic AV outflow flow jet patterns and wall impingement zones which correspond to variable WSS eccentricity and regional peak systolic WSS between different valve groups.

